# Role of cGMP/PKG in fat tissue

**DOI:** 10.1186/1471-2210-11-S1-O35

**Published:** 2011-08-01

**Authors:** Alexander Pfeifer

**Affiliations:** 1 Institute of Pharmacology and Toxicology, University of Bonn, 53105 Bonn, Germany; 2 Pharma-Center, University of Bonn, 53113 Bonn, Germany

## Background

With more than half a billion adults world-wide being obese, obesity has reached pandemic dimensions. Thus, regulation of fat cell differentiation and function is of great interest for pharmacological research.

The cyclic GMP (cGMP) signaling cascade plays an outstanding role in the cardiovascular system. Several studies demonstrate that cGMP signaling plays also an important role in fat tissue. Interestingly, essential components of the cGMP pathway have been found in both white (WAT) and brown (BAT) adipose tissue. White adipocytes mainly store energy in form of lipids, whereas brown fat cells dissipate energy in form of heat.

## Results

Apart from soluble and particulate guanylyl cyclases that produce cGMP after stimulation with nitric oxide or natriuretic peptides, respectively, fat cells express different cGMP receptors. Three major types of cGMP receptors exist in mammals: cGMP-dependent protein kinases (PKGs), phosphodiesterases and cyclic nucleotide gated channels.

PKGs are mediators of cGMP effects in a broad spectrum of tissues. PKGI plays a major role in the cardiovascular system including smooth muscle and platelets, whereas PKGII mediates cGMP effects in the skeletal system (cartilage and osteocytes) and the intestinal mucosa.

PKGI is expressed in both WAT and BAT. Using gain- and loss-of-function models, we and others have unequivocally shown that PKGI plays an important role in brown fat cells. We showed that ablation of PKGI suppresses differentiation of brown fat cells in vitro and in vivo, whereas the Nakao lab demonstrated that overexpression of PKGI results in increased mitochondrial biogenesis, enhanced expression of adipogenic markers in BAT and prevents obesity.

Concerning the role of PKG in WAT, it was previously shown that PKGI is expressed in human white adipocytes and that it mediates ANP-induced lipolysis. We found that PKGI is also expressed in white adipocytes of mice and recent data show that PKGI is also important for differentiation of white adipocytes.

## Conclusion

Taken together, cGMP/PKGI signaling plays an important role in both white and brown fat cells.

